# Current Consumer Perceptions of Animal Welfare across Different Farming Sectors on the Island of Ireland

**DOI:** 10.3390/ani12020185

**Published:** 2022-01-13

**Authors:** Sharon Sweeney, Áine Regan, Claire McKernan, Tony Benson, Alison Hanlon, Moira Dean

**Affiliations:** 1 Department of Agri-Food Business and Spatial Analysis, Rural Economy Development Programme, Teagasc, Mellows Campus, H65 R718 Athenry, Ireland; sharon.sweeney@teagasc.ie; 2 Faculty of Medicine, Health and Life Sciences, The Institute for Global Food Security, Queen’s University Belfast, Belfast BT7 1NN, UK; c.mckernan@qub.ac.uk (C.M.); t.benson@qub.ac.uk (T.B.); moira.dean@qub.ac.uk (M.D.); 3 School of Veterinary Medicine, University College Dublin, D04 V1W8 Dublin, Ireland

**Keywords:** attitudes, beliefs, citizen, farmer, labelling

## Abstract

**Simple Summary:**

The well-being and welfare of animals on farms is a topic of interest to many people. On the island of Ireland, this issue has received increased attention because of political and governance changes, including Brexit and COVID-19. Policy-makers and industry are considering labelling schemes to inform the food consumer about welfare standards on farms. Focus groups and an online survey were carried out with members of the public on the island of Ireland to explore their awareness, perceptions, and attitudes toward farm animal welfare standards on farms in Ireland and Northern Ireland. Most consumers believed farm animal welfare standards were high, although different farming sectors were rated differently: beef and dairy farms were viewed more positively, and pig and poultry farms were viewed less positively. The living conditions of the animal, size and intensity of the farm, national standards and schemes, and visibility all influenced perceptions of welfare standards. The public also expressed a lack of knowledge and information on the topic. In developing new policies and labelling schemes, it is important to be apprised of the current awareness, attitudes, and perceptions that the public has regarding farm animal welfare standards, as identified in the current paper.

**Abstract:**

There has been increased public interest and concerns in issues such as farm animal welfare (FAW) on the island of Ireland, stoked in part by political and governance changes, such as Brexit and COVID-19. Front-of-pack food labelling represents a primary information channel for many people. In advance of considering formalised food labelling schemes, specifically relating to FAW, it is important to ensure an up-to-date understanding of current consumer perceptions of FAW. With this aim, the current study utilised a mixed methodology. Nine focus group discussions (*n* = 41) and an online survey (*n* = 972) with food consumers in Ireland and Northern Ireland explored perceptions of FAW. Results suggest that overall perceptions of FAW are high, and consumers perceive FAW to have improved in the last decade. Quantitative (ANOVA) and qualitative results show variations in perception of FAW between sectors. Results from the focus group discussions identified factors underlying consumers’ perception of FAW: the living conditions of the animal, size and intensity of the farm, national standards and schemes, and visibility. Information insufficiencies and knowledge gaps were identified. The findings are discussed in relation to policy implications for the role of public engagement, front-of-pack welfare labelling, and quality assurance schemes.

## 1. Introduction

Agriculture has played a historically important economic, social, and cultural role on the island of Ireland (IOI), and farms have long been central to the socio-economic vitality of rural communities. The landscape of farming, society, and food on the IOI has undergone many changes in the past few decades, with agriculture seeing increased intensification and specialisation of production [1]. The IOI, in line with many modern industrialised countries, has seen a shift towards increased urbanisation and higher wealth [2], contributing to a growing disconnect between consumers and farming. Consumers are increasingly reliant on third parties and trust-based processes (e.g., quality assurance schemes, labelling) to find out about farming and food production methods. The last few decades have seen a heightened awareness of food choices and decisions by consumers. Particularly, considerations of farm animal welfare (FAW) have begun to feature strongly in consumer decision-making [3]. Modern consumers are becoming increasingly judicious, with growing expectations. The last decade has seen a mounting number of consumers actively pursuing products with ethical, sustainable, and transparent production methods [4]. This is also noted by the growth of niche to mainstream consumer segments such as veganism, ethical consumerism, and flexitarianism [5]. This shift in consumer values is facilitated, in large, by multi-directional information flows and the sharing and discussion of information, images, and videos online and via social media [5,6]. The rising momentum of food bloggers and online influencers create increasingly informed and networked consumers who seek out social proof of food decisions. 

Political and governance changes around Brexit and COVID-19 have also had implications for how the agri-food sector is perceived by the public. UK consumers report Brexit concerns around food quality with lower animal welfare standards potentially entering the market [7]. Furthermore, COVID-19 has led to increased awareness amongst consumers on the IOI regarding OneHealth concepts and animal welfare information on food labels [8]. The last decade has seen significant non-regulatory initiatives in the area of animal health in Ireland [9]; the next decade will see changes in farm animal health practices in line with new EU legislation in the area of veterinary medicines [10,11], in efforts to tackle the global threat of antimicrobial resistance. Despite significant changes to animal health practice in the farming sector, no standardised front-of-pack labelling system solely dedicated to FAW in Ireland or the EU exists. In the context of these macro-changes in the governance and production of food, it is of interest to consider the current climate of consumer perceptions of FAW on the IOI and what this may mean for food policy in the area of public information and labelling in the future.

Up-to-date research examining consumer perceptions and attitudes specifically towards FAW on the IOI is scarce. Within the Republic of Ireland (ROI), early research showed mixed results. In the wake of the BSE crisis, consumers had deep concerns regarding the welfare of animals in agriculture and reported animals receiving appropriate feed as their highest welfare concern, reducing their consumption of animal products accordingly [12]. However, when viewing FAW from a perspective that did not consider the issue of animal feed, research found welfare issues were not a major driving influence of Irish consumers’ meat purchasing decisions [13]. Recent literature shows that the public perceives a lack of knowledge on farming and practices. Regarding FAW, they feel there is not enough information available and report a wish to have more [14,15]. Irish consumers overwhelmingly felt it was “very important” (80%) or “somewhat important” (17%) to protect the welfare of farm animals [15]. Northern Ireland (NI) data (aggregated within UK data) report similar figures of 78% and 20%, respectively [15]. An earlier report [16] asked consumers to rate the same on a scale of 1 to 10 (1 = not at all, 10 = very important). At that time, the average ratings were 8 (Ireland) and 7.8 (UK), indicating relatively stable prioritisations over time and geographical region. The importance of animal welfare to consumers is not just confined to ethical motivations; research in Ireland has shown that high FAW provides a cue to consumers that the product is healthier, safer, and of higher quality [5,12]. 

Alongside rating FAW as highly important to protect, a similar majority of Irish (80%) and UK (76%) respondents believe that farm animal welfare should be better protected than it is presently [15]. This could suggest a negative perception of conditions on IOI farms, but in the absence of this data, it is unclear what perceptions consumers currently hold. Research from other geographical settings indicates that consumers’ views about FAW vary across different production systems and farming sectors [17,18,19,20]. Consumer’s welfare attitudes, in general, are shown to be more negative towards modern, higher intensity farming [14]. Greater concerns arise around poultry farming, lower concerns for dairy, and opinion on the production of pork is mixed [14]. These sector differences are consistent with earlier Irish and UK data [21]. However, there are concerns about changing public perceptions on welfare issues in different sectors, including the dairy sector, as specific practices (e.g., calf-cow separation, male dairy calves) are increasingly discussed in the public domain [22,23]. 

Fraser et al. [24] define animal welfare based on the three-sphere model: the animal’s normal biological functioning (includes ensuring the animal is healthy and well-nourished); natural living (includes ability to express normal behaviours), and; affective states (includes the absence of negative emotions, such as pain or chronic fear). Consumers’ own definitions of ‘good’ FAW inform the factors that shape their perceptions of FAW [12,25]. While some argue that the growing disconnect and lack of knowledge consumers have about agricultural practices are the reason for negative welfare perceptions, values play a particularly important role in shaping consumers’ views [26]. Previous research in Ireland has shown that for consumers, living a natural life is an important element of FAW [12], and this is consistent with the wider body of research [14,26,27,28]. Within “naturalness”, consumers see outdoor access as an important welfare consideration together with the animal having enough space [12,14]. Factors, such as additives, unnatural foods, and hormones were also of great concern in Ireland [12] and beyond [14,25]. That consumers hold different levels and types of concerns highlights a heterogeneous group when it comes to defining positive FAW from the consumers’ perspective. Younger age groups, females, higher social class, and pet ownership have been linked to higher FAW concerns. Meanwhile, living in a rural location, having previously worked or visited a farm, or having regular contact with farmers were associated with less concern about FAW and a greater acceptance of modern farming practices [14]. 

There has been increased policy focus on the role of animal welfare in agriculture in recent times [29,30]. One strategy being explored is the role of quality assurance schemes and welfare-specific food labels [31]. Currently, the only EU-wide obligatory system of labelling relating to animal welfare exists for eggs. This label defines different production methods (cages, free-range, barn, etc.) and is based on EU legislation. EU organic production rules on livestock include respect for animal welfare [32]. Prior to the introduction of any higher welfare labelling strategies, it is necessary to understand consumers’ awareness and motivation to engage with the issue of farm animal welfare [33]. The current study presents a mixed-methods exploration of current consumer perceptions of FAW on the IOI, a microcosm exploration in which the findings will also point to implications for other European countries. Using quantitative methods, the study will provide a measure of current consumer perceptions of FAW, and consumer perceived changes in FAW over the last decade. Using qualitative methods, the study will then provide an in-depth explanation of the factors shaping and driving these currently held perceptions. The implications of the findings for policies on public engagement, food labelling, and quality assurance schemes will be discussed. 

## 2. Materials and Methods

Current consumer perceptions of FAW on the IOI were explored using a mixed-methods approach allowing for a detailed and triangulated exploration of consumer perceptions of animal welfare across different farming sectors on the IOI. The data for this study were collected as part of a larger project investigating consumer perceptions of FAW, antimicrobial use and antimicrobial resistance within agriculture.

### 2.1. Survey

#### 2.1.1. Method

The current study draws on survey items from a larger survey project that explored consumer perceptions of FAW and farm animal health. A cross-sectional online survey was administered to food consumers in Ireland and Northern Ireland (*n* = 972). The survey included 24 survey scales that explored consumer perceptions of FAW, and knowledge and attitudes towards the use of antimicrobials in farming. The current study reports the findings from two variables included in the survey to capture current consumer perceptions of FAW. To measure consumer perceptions of FAW, two questions were adapted from previous survey research [33]. (i) “Thinking about farms in Ireland <Northern Ireland>, how do you rate the animal welfare conditions in the following sectors?” [beef production, chicken production, egg production, pork production, and dairy production] using a five-point Likert scale consisting of (1) very poor–(5) very good. (ii) “In general, over the past 10 years, do you think that farm animal welfare in Ireland <Northern Ireland> in each of the following sectors [beef production, chicken production, egg production, pork production, and dairy production] has…” using a five-point Likert scale: (1) gotten much worse–(5) gotten much better. An option of “I don’t know” was included alongside each scale. A market research agency was used to recruit participants and administer the survey. Data collection took place during September 2020.

#### 2.1.2. Sample

A quota sampling procedure achieved a sample representative of gender, age, region (urban/rural), and social class. Inclusion criteria included: aged 18+, a consumer of either meat or dairy, and at least partial responsibility for household grocery shopping. Individuals who reported their occupation as ‘farmer’ were excluded from participation. The socio-demographic breakdown of the sample is provided in Table S1. 

#### 2.1.3. Procedure

Prior to commencing the online survey, participants received information regarding the study and consent was obtained. In total, the survey took participants approximately 10 min to complete. Upon completion of the survey, participants were debriefed and provided with websites where they could seek further information on farm animal health and welfare. All participants received an incentive through the market research agency for taking part. 

### 2.2. Focus Groups

#### 2.2.1. Method

Nine focus groups, with between 3 and 6 participants (*n* = 41), were conducted across the island of Ireland in October 2019 to explore consumer perceptions of FAW. Focus group discussions were chosen to facilitate the exploratory nature of this stage in the study design. They provided a mechanism whereby a deeper understanding of consumer perceptions of animal welfare could be explored within the broader social context from which the participants originated.

#### 2.2.2. Sampling and Recruitment

A purposive sampling method based on age, gender, urban/rural, socio-economic status, and parents was designed to ensure participants were selected to reflect a range of the total study population. A vegetarian group was included as they are reported to have higher awareness and greater involvement in animal welfare issues [34]. Recruitment took place through poster and flyer advertising, convenience sampling, and engaging community groups. The criteria for recruitment alongside the location of the focus groups are presented in Table 1. Each participant received a EUR 50/GBP 45 voucher as incentive for taking part.

#### 2.2.3. Procedure

The focus groups followed a semi-structured discussion guide [35] that was developed by the research team. Once complete, the guide was pilot tested in a convenience sample. Because no major revisions had to be made to the FAW discussion guide, the pilot discussion results were included in later analyses. The final guide was anchored around the general perception of FAW on the IOI and related consumer behaviour. Participants were asked open questions about their perceptions of welfare on farms and not about specific practices, e.g., “I am interested in discovering what comes to your minds when you think about how animals are looked after on (Northern) Irish farms?” followed by prompting specific sectors if necessary, e.g., “What about poultry farms, specifically?” This facilitated the freedom to introduce any farming practices participants perceived as negative or positive drivers of FAW. In order to stimulate discussion around their behaviour as consumers, the participants were then mentally situated within their normal shopping environment before being asked questions such as “When buying <pork> products, what is important to you, what do you look for?”

At the outset, a written explanation of the study aims was given to participants alongside information outlining steps taken to ensure confidentiality and anonymity. These were read aloud by the facilitator to ensure comprehension. Once consent was established, participants completed a short demographic questionnaire: gender, age range, children in household, household income, pet ownership, and farm visits (Tables S1 and S2).

The focus group discussions were 1½ hours in length. A note-taker was available for NI groups. To maximise the efficacy of the focus groups, various strategies were adopted. Firstly, everyday language was used to ensure all participants could fully engage with the topics. Secondly, a range of food product labels were available for use as prompts, where necessary, to stimulate conversation and as an aid in situating the consumer within the context of their normal purchasing environment. The discussions were audio-recorded with participants’ full knowledge and permission, and anonymised transcripts were prepared for analysis.

### 2.3. Data Analysis 

All analyses of the survey data were conducted using IBM SPSS Statistics for Windows, Version 24.0. Frequency statistics provided an overview of consumer perception of FAW and consumer perception of improvements of FAW over time for each sector. One-way repeated-measure ANOVA’s were used to compare mean scores across sectors (“I don’t know” response listwise deleted) to investigate differences in (i) consumer perceptions of FAW between each farming sector and (ii) consumer perceptions of changes over time between each farming sector. 

Data from the focus groups were analysed using an inductive thematic approach. This consisted of data-driven coding to distinguish overall themes, followed by further, in-depth, interpretive coding using NVivo 12. To ensure the reliability of data interpretations, analyses were performed through the collaboration of two researchers.

## 3. Results

The findings from the survey and focus groups are presented together in the following sections to provide a triangulated perspective on public perceptions of farm animal welfare on the island of Ireland. 

### 3.1. Perceptions of Farm Animal Welfare on the Island of Ireland

Frequency statistics from the survey provide an overview of current consumer perceptions of FAW (Table 2) and consumer perceptions of change over the last ten years (Table 3) within each of the sectors. Findings for each region (NI and ROI) are provided in Table 2 and Table 3. Similarities of FAW perception were seen across the regions. However, more NI respondents rated welfare in poultry meat and egg production positively and less rated them negatively compared to ROI respondents. On both questions, a higher proportion of NI respondents selected “don’t’ know” across all of the sectors compared to ROI respondents.

The majority of respondents in the survey reported that they perceive positive conditions (“good” or “very good”) across all of the sectors (Table 2). A smaller proportion of respondent’s rate conditions as “poor” or “very poor” for any of the sectors. Dairy production had the highest proportion of positive ratings (73.1% rated the sector as “good” or “very good”) and lowest proportion of negative ratings (3.6% rated the sector as “poor” or “very poor”). Consumer ratings for beef were similar to dairy; 71.7% rated welfare in the beef sector as “good” or “very good”, and 4.5% rated it “poor” or “very poor”. Pork and egg production showed similar positive ratings with 52.4% and 53%; however, egg production showed a higher negative rating with 13.8% compared to 10.9% for pork. Compared to the other sectors, chicken production had the least amount of positive welfare ratings (44.4%) and the highest amount of negative ratings (16.7%). Between 14% and 27% of all respondents rated FAW as moderate across all of the sectors.

Most respondents in the survey reported that they thought FAW had improved rather than worsened across all sectors over the last ten years (Table 3). The largest proportion (32–36%) believe FAW has improved “somewhat” across the sectors. The greatest proportion of positive change was seen for dairy (62.7%) and beef (59.9%), followed by egg production (54.3%), chicken production (49.8%), and pork production (48.4%). The greatest proportion of negative response scores was seen for chicken (11.9%) and pork (9.6%), followed by egg production (8.5%), and both beef and dairy production (4.7%). Between 23% and 28% of respondents think FAW has remained about the same.

Similar to the survey findings, the focus groups also found that participants’ initial views on how animals are raised on IOI farms were mostly positive. Based on the focus group discussions, the image of IOI farms is one of traditional farms, pasture-based, with very few immediate concerns around welfare. Participants did not believe inhumane treatment occurred and that any incidences of animal neglect were confined to isolated incidents. When probed on specific sectors, concerns about specific farming practices did, however, emerge. This mirrors the sector-specific survey findings. The next section considers these sectoral differences in more detail.

### 3.2. Perceived Farm Animal Welfare across the Different Farming Sectors on the Island of Ireland

Using the survey data, a one-way repeated-measures ANOVA was conducted to test if the differences we observed in consumer perceptions of welfare between the production sectors significantly differed. A Mauchly’ test indicated the assumption of sphericity had been violated, χ^2^ (9) = 377.09, *p* < 0.05; therefore, degrees of freedom were corrected using Huynh-Feldt correction estimates of sphericity (ε = 0.79) [36]. The results show there was a significant effect of sector on consumer perception of FAW, F (3.16, 2443.59) = 218.68, *p* < 0.001, ηp^2^ = 0.22. A post-hoc analysis using a Bonferroni adjustment shed light on the particular sectors that significantly differed (Table 4). The mean difference was largest between chicken and dairy production (0.71), which represented the lowest and highest means scores, respectively. No significant difference was found in the level of perceived FAW between dairy and beef production, with both showing similarly high means scores. However, egg production had a significantly higher perceived FAW mean score compared to chicken production, suggesting the consumer perception of the treatment of hens is comparably different between the production sectors.

A second one-way repeated-measures ANOVA was conducted to test if consumer perceptions of welfare change over the last ten years differed between the beef, chicken, egg, pork, and dairy production sectors. The Mauchly’ test indicated the assumption of sphericity had been violated, χ^2^ (9) = 157.14, *p* < 0.05; therefore, degrees of freedom were corrected using Huynh-Fedlt estimates of sphericity (ε = 0.91) [36]. The results show there was a significant effect of sector on consumer perception of FAW improvement over the last ten years, F (3.62, 2891.03) = 58.56, *p* < 0.001, ηp^2^ = 0.07. A post-hoc analysis using a Bonferroni adjustment (Table 5) informed us that the mean difference in ratings of change was largest between chicken and dairy production (0.31). No significant difference was found in the level of perceived FAW improvement between the beef and dairy production systems. The results suggest that consumer’s regard egg production to have improved significantly more than chicken production. Consumers’ perception of FAW change in pork and chicken production does not significantly differ, suggesting that consumers rate them similarly for change in the last ten years.

Aligned to the survey findings, in the focus groups, variation in attitudes toward IOI farming emerged when participants were probed on specific animal categories, i.e., cows, chickens, and pigs. These are discussed in more detail below and summarised in Table 6. 

#### 3.2.1. Cows

Positivity emerged for sectors concerning cows; this was particularly the case for participants with proximity to these types of farms. These participants reported seeing the cows and/or the farmer and being reassured by this.

*“P1-Cows are in the field all around me and you’d see the farmer coming to feed them*

*P2-They seem to be well looked after [multiple participants agree]”*

—Seniors Rural

The few FAW concerns that did arise involving cows were specific to dairy production. These concerns related to the quality of life of the animal, which has to “*produce milk for the majority of the year*”, and also included concerns for the health and welfare of the cow due to “*forced impregnation*” and “*cow-calf separation*”. All dairy buying participants reported that FAW rarely entered their consciousness when buying dairy products, so it had little influence on their buying behaviour. Instead, participants were more interested in the freshness and the origin of the milk they buy, mainly preferring local dairies. The effect of the BSE crises still lingered with a small number of participants, predominantly within the senior groups, some of whom have not eaten beef since.

#### 3.2.2. Poultry

The poultry sector emerged as the sector with the greatest concern for FAW within and across all groups. The concerns that arose predominantly centred on the living conditions for the animals. Questions also emerged around the use of antibiotics and growth-promoting hormones. Participants perceived cramped and unhealthy conditions, with hens “*never seeing the light of day*”. There was a higher awareness of differing production systems within this sector, with some trust issues emerging around the standards needed within them. A noticeable preference for free-range eggs was seen, which translated into purchasing behaviour. The higher welfare egg buying behaviour, seen in the majority, was driven by welfare concerns, a smaller price difference, and a perception of higher quality. The short food supply chain link was apparent in egg buying behaviour, with some participants preferring to buy eggs directly from suppliers with “*garden hens*”. With regard to free-range or organic chicken meat, the translation of concern into purchasing behaviour was less strong. A larger price difference was cited as a barrier to purchasing this product; however, those who did purchase free-range or organic chickens saw them as a worthy investment.

*“P1-I think it’s because I hate the thought of the chickens in a barn all stuck together… [P2 agrees]…and looking absolutely woeful*

*P3-yes we’ve seen those pictures, haven’t we? [P2 agrees]*

*P1-and it’s just so awful..[P3 agrees]..that I can’t bear the thought of contributing to that way of farming by buying it”*

—Seniors Urban

Perceptions of different motivations driving farmers’ behaviours also shaped consumer perceptions of welfare issues in poultry farming. For example, the young rural males’ group discussed the role that pride and stigma can play in shaping how well farmers take care of their animals, perceiving this to be stronger in some sectors (e.g., cows) than in sectors such as poultry where the farming system was perceived more as a large corporation-type business than a family farm-run enterprise.

*“P1-You would assume as far as welfare is concerned, if it’s not free-range, it’s probably not good living for them like.*

*P2-Definitely you would be more concerned about poultry*

*P1-Yea, as [P4] mentioned, it’s the pride like. I don’t think there would be pride in chicken farmers like there would be in the cattle farmers*

*P3-no, no there wouldn’t.*

*P4-They are boxed away. Nobody sees…*

*P2-It’s just mass consumption like. You are not taking your chickens to the mart like!*

*P4-There is 20,000 chickens in a shed in Limerick or Monaghan and all of a sudden they are just brought and processed. Whereas if John Joe down the road is going to the mart and he is bringing 15 bullocks in, you can be sure there is 5 or 6 lads looking and going what state are they in or what are they like.*

*P1-it’s going to be talked about.*

*P4-Absolutely. Whereas nobody is going to talk about the chickens.*

*P3-you might have someone taking a bit of pride in eggs but that’s just because the hens are going to be about the yard picking.”*

—Young Adult Rural

#### 3.2.3. Pigs

The pig sector emerged as the sector where participants had the least amount of knowledge across groups. Consumers were cognisant that although their knowledge of FAW was low overall, it was lowest for the pig sector. Many consumers were concerned that they never saw pigs; the perceived hiddenness of this sector led to questions and suspicion. Concerns that did arise within the groups centred mostly on the living conditions and housing of pigs. Participants described cramped, indoor conditions.

*“P1-And I remember we were even brought on a school tour […] I suppose they were kept kinda very… erm..*

*P2-Enclosed*

*P1-Enclosed like in a small amount of space for such a big animal… [multiple participants indicate agreement with this]… but erm yeah like when I think about it now I think “ooh god”*

*P2-I’d be sorry for the pig*

*P1-I don’t remember ever seeing them out*

*P3-No they are always in enclosed areas*

*P1-I think that it is interesting that we eat such a huge amount of erm you know pork and pig…[P2 & 4-We do]…Like we nearly eat it every day especially around breakfast time. And you never see a pig, like it’s very strange isn’t it. We don’t see pigs anymore, no”*

—Mixed Age Female

Participants described pigs as being sentient creatures, and as such, they elicit more welfare concerns. There was great concern amongst some participants related to the slaughtering process and awareness of the animal around this, especially within the vegetarian group. However, insight into any level of the food chain helped to build trust, which was demonstrated by one participant within that group who had visited a pig abattoir; this reassured him that the high standards he saw there would be translated to farm level. When it came to behaviour within the current study, despite the high level of concern found, participants reported not normally thinking about the welfare of this animal when purchasing pork products.

### 3.3. Knowledge Gaps and Heuristics Used to Make Judgements about Farm Animal Welfare

As seen in Table 2 and Table 3, a sizeable proportion of survey respondents answered, “I don’t know”, when asked to judge farm animal welfare standards of the different sectors. The pork sector provoked the greatest response of “don’t know” in the perceptions of FAW (15.6%) and change in FAW over the last ten years (14.1%). Chicken and egg production had similar amounts of “don’t know” in both questions (11–12%). The dairy and beef sector had the lowest proportion of “don’t know” (9–11%).

In the focus groups, knowledge about farming was also expressed to be low, with the exception of young rural adults who had the highest proximity and personal connection to farms due to living in a rural community and having friends and extended family members who were farmers. Participants themselves acknowledged they were low in knowledge about farming practices. Without knowledge or experience of farms, participants used a number of heuristics to help them make judgements about FAW. A number of factors shaped their perceptions of positive and negative FAW. 

#### 3.3.1. Living Conditions of the Animal

When it came to defining positive welfare, variation was found within the discussions; however, participants predominantly defined FAW in terms of the living conditions of the animals. Terms strongly related to good FAW and seen to be very important included “outdoor access” and “having space”.

*“P3-What I think is, is that they’re not locked up or anything, they are out in green fields [P4 agrees]*

*P2-That was my one…I felt the same yep*

*P5-Yeah. Animals outside … in bad weather they kind of go inside, you see them in [through] the hedges.”*

—Seniors Rural

#### 3.3.2. Size and Intensity Level of the Farms 

One of the primary factors in how consumers distinguished between FAW related to the perceived size or intensity level of the farm. Unprompted, consumers made the distinction between intensive and extensive systems. IOI farming was primarily identified as an extensive system. Smaller farms were seen as the norm and as the ideal, with higher FAW and farmers taking pride in their animals. Concerns that arose within the discussions were mostly directed at large farms. The industrialised system was linked with too many animals on the farm and more “factory-like” conditions contributing to greater FAW concerns. Consumers saw intensive systems as having less regard for the individual animals and as being more “business-like” and profit-driven, resulting in a cheaper, lower quality product. Participants acknowledge that market demand is driving intensive systems and discussed the need to change their consumption patterns and reduce the amount of meat they eat. Many participants were supportive of farmers, associating them with small profit margins and hard work. They felt farmers should be compensated for any improvement costs. They believed markets and industry were putting a lot of pressure on farmers to increase productivity while driving prices down. They talked about the need to pay more for better quality leading to less pressure on the farmer and better practices on the farm.

*“P1-like cheap meat, … it’s the general consensus that it probably isn’t that good quality.*

*P2-Yeah… I suppose something I would often consider is the pressure perhaps the farmer is under to meet financial targets. And as a result I suppose the animals are then under pressure because… we all read in the newspapers and in the media about the farmer being squeezed out a little bit and getting a very small percentage of the price of the milk that we buy. So, you know, that must have an impact on what happens in some of the larger scale farms”*

—Young Adult Urban

As a citizen, participants feel they have a responsibility for FAW, but as a consumer, their decisions are still ultimately often driven by other factors. When making purchasing decisions, consumers cite attributes such as price, taste, and perceived quality as their main drivers. The main barrier to purchasing higher FAW products was the perceived cost of such products. The majority of the consumers were price-conscious, with the additional price seemingly prohibitive; however, some held a strong suspicion of cheap food, which was equated with intensive systems and lower quality, especially for the young adult urban group. 

#### 3.3.3. National Standards and Schemes 

Within all of the focus groups, consumers felt IOI farms had higher standards of welfare and produced superior quality goods compared to non-IOI products. This was also seen amongst the vegetarian group; of note, this group were not vegetarian solely for animal welfare concerns but also due to sustainability and environmental concerns. The farm-connected young rural adult group trusted that the standards and regulations in Ireland ensured FAW was a non-issue. Broader participant discussions around labels highlighted the complexity faced in assessing the differing labels, certification schemes, and regulation standards. Consumers believe it is the role of supermarkets and the government to inform them and make choices available; however, views on using labels to make judgements on FAW were conflicted. When discussing labelled information on how animals are reared, the majority immediately say they look for signs about proof of origin, “*the Bord Bia sign*”; the “*Guaranteed Irish*” sign; “*the little Irish sign*”. This belief in the quality of Irish produce translated into participants making purchasing decisions based on the origin logo. Within the NI discussion of labels, the red tractor logo (UK) had a low level of awareness and understanding, ranging from complete unawareness to some recognition that it is a quality mark. A small number of participants, predominantly in the Parent Urban group, never look at any labels. Of consumers that did attend to labels, many reported their trust in labels to be low. They reported not making purchasing decisions based on labels due to this mistrust, which was driven by confusion around what the labels actually represented and the standards for this. Even within the preferred Irish label, mistrust arose around its genuineness, with some believing that if any amount of processing (e.g., packaging) was done in Ireland, it would allow foreign products to be labelled as Irish.

*“P1-Well that says Bord Bia quality assurance and there was a time that that might have been enough but that’s not enough for me now for me to trust it*

*P2-that’s it*

*P3-and the origin Ireland; that can be…*

*P2-Where it is packaged*

*P3-Say if that’s chicken, yeah, that if that chicken was deboned or one bone taken out of it in Ireland then its origin is Irish*

*P1-Right OK, I didn’t know that*

*P3-it’s a bit of a scam*

*P2-I think this isn’t worth the paper that it is printed on”*

—Parent Rural

There was limited vocalisation of support across the groups for ethical or welfare-driven labelling. A small minority were against the idea completely, seeing it as “*pointless*”, especially within the young rural adult group who felt that the standards in place were high enough. Participants wanted to increase their level of trust through greater transparency around the origin of their food, production processes, and the standards that need to be met for labelling. 

#### 3.3.4. Visibility and Closeness to Farms

Visibility, i.e., being able to see animals in fields and seeing farmer-animal interaction, was used as a strategy by consumers to reassure themselves that animals were well looked after. This was most commonly the case for pasture-based animals such as cows and sheep. Where participants perceived a lack of visibility, suspicion was aroused. If animals are not visible out in the fields, then the perception is that they are being kept indoors, creating images of crowded conditions and of a more intensive system.

*“P3-…You just don’t see what’s going on with the chickens do you?*

*P2-When you think about it, you can see cows in a field. But chickens I suppose are in a coup so you are not really seeing them, kind of that whole behind closed doors thing.*

*P4-Close enough to where I am living, there is a fella rearing turkeys and I saw them out there the other day and I know that’s a quality product he is producing.*

*Moderator- Why do you think that?*

*P4-because seeing, I saw it! I saw them. There was 30 of them looking out over the fence at me!*

*P3-yea, it’s what you see isn’t it?*

*P4-whereas the chicken farm, when I used work down the country I used pass 2 or 3 chicken farms on the way to work. They were far off the road, not that you would want to pull in, but you would never see anything going on there you know what I mean?*

*P2-They are almost hidden in a way? Therefore then, that is nearly enticing them to cut corners.*

*P1-Or assume they are cutting corners. They mightn’t be at all.*

*P2-well if it’s hidden like, and the chickens are kept inside…”*

—Young Adult Rural

A preference for short supply chain production systems was seen within the focus groups, especially supply chains that involve direct contact with the farmer. The close connection to the farmer and belief about the type of relationship the farmer has with their animals invoked feelings that the animals on these types of farms were well looked after. The premium price that was paid for these products, although unaffordable for some, represented purchasing high-quality products with a “story”.

## 4. Discussion

This study sought to explore current consumers’ perceptions of FAW and perceived changes in FAW over the last ten years on the IOI. Alongside quantitative exploration, focus group discussions provided additional insight into some of the main factors underlying these perceptions.

### 4.1. Current Perceptions of Farm Animal Welfare on the Island of Ireland

The evidence suggests that negative consumer judgements of FAW on the IOI may have reduced over time. Firstly, the results of the survey show that a larger proportion of consumers believe FAW in the last 10 years has improved rather than worsened. Secondly, when the two negative (lowest) responses on the perception of FAW are combined and compared with the data available from UK and Ireland in an earlier European attitudinal survey [21], both surveys present an insight into public perceptions of FAW in different sectors from different time periods. Despite differences in the questions and scales, the large percentage differences seen in the comparison does suggest that public concerns about FAW may have reduced over time within the egg, dairy, and pork production sectors (Table 7). Table 7 provides a descriptive comparison of the survey findings only. No previous survey data was found by the authors for chicken and beef in both jurisdictions. 

Results from the focus group discussions and the survey show variation in the perception of FAW across sectors. Greater concerns arise for FAW in poultry and pork production, lower concerns appear for dairy or beef. The patterning of concern across these sectors remains consistent with that found in earlier and wider literature [12,14] and in other geographic regions [17,18,19,20]. Greater concerns arose for more intensive systems, seeing them as more factory-like with less regard for individual animals [12,14,18]. Visible pasture-based systems and direct contact with farmers in a short supply chain reassured consumers that the animals on those farms were well treated. Short supply methods represent a more traditional type of food supply in a highly visible and local supply chain [37].

### 4.2. Information Insufficiencies and Knowledge Gaps

While overall, relatively positive judgements were held by many of the public, there was evidence that information insufficiency was high, i.e., consumers perceive they lack adequate information on FAW. When asked on the survey for their judgements of FAW, a sizeable proportion of respondents answered “Don’t know”. Those who did provide a rating may or may not have high knowledge of FAW; however, we can reasonably ascertain that for those who chose the “don’t know” option, they felt that their knowledge was too low for them to make a judgement [33]. Findings from the focus groups showed consumers were cognisant that their knowledge of farming and FAW was low. A recent Irish consumer trend report found “people want to be informed about the animal products that they purchase and will respond to claims that instil trust and confidence” [38]. The prevalent unavailability and subsequent trust of information sources may be contributing to a gap between the consumer and agriculture [39]. A 2016 Eurobarometer reports 65% (ROI) and 48% (UK) of respondents would like to have more information about the conditions in which farm animals are raised [15]. Given that early research in Ireland also reported a lack of subjective knowledge [12,16] and a wish to be better informed [12], more may need to be achieved in effectively addressing this consumer concern. 

Evidence of a consumer-farming disconnect was also apparent in the *types* of welfare issues raised by consumers compared to those raised at the farm and research level. When looking at the results of this study through the three spheres of welfare [24], the concerns consumers express around animal welfare differ in many ways from the issues being prioritised within agriculture. The qualitative findings in this study revealed that consumers tended to discuss welfare issues predominantly related to natural living and affective states, e.g., “outdoor access”, “having space”, etc.; these are similar to the concepts found in other studies [27,28]. Where natural behaviours are perceived to be met, such as within the pasture-based systems, the consumer tended to focus on affective issues such as quality of life for the animal and distress from practices such as cow-calf separation. In contrast, good FAW for farmers generally aligns most often with satisfying the biological function of an animal, followed by the affective state of an animal, and thirdly, the ability of an animal to engage in natural behaviour [40]. This phenomenon was noted in the previous literature [14,23,25]. Recent research on the perceptions of dairy farmers in Ireland shows their concerns for calf welfare [41], especially the issue of surplus male dairy calves [22,42]. These concerns did not spontaneously arise during the focus group discussions in the current study, indicating they may not currently be at the forefront of the public’s mind. 

Within this study, dairying was perceived as extensive and having a high level of animal welfare; research shows that this sector has become increasingly intensive with dairy herd expansion, compact calving, and zero-grazing systems [43]. Selective breeding of high-yielding cows has corresponded to a high prevalence of production diseases such as lameness, mastitis, metabolic disorders, and reduced fertility, culminating in premature culling [44]. Skin damage during housing, tail injuries and nasal health, and lameness were seen to be needing improvement [45]. Painful procedures, such as disbudding, dehorning, and castration, performed with absent or inadequate pain relief are evident in dairy [46] and are also relevant to beef farming [47]. Regarding intensive poultry and pig production systems, the reservations expressed by participants in this study are mirrored by scientific evidence [48,49]. However, tail biting is one of the primary welfare problems in pig production, and this did not arise within the consumer discussions. Tail biting can lead to injury, pain, stress, and the use of antimicrobials, which feeds into increased antimicrobial resistance. Tail docking, used to tackle tail biting, is also acutely painful and is known to cause long-term sensitivity in the tail. It is not always effective against tail biting and is banned by EU regulation; however, adherence is shown to be poor across Europe, e.g., 99% of Irish and NI pigs are docked [50]. Solutions geared toward the reduction of tail biting and tail docking may lie in the consumer-preferred “natural living” sphere as tail biting is seen to be a misdirection of a pigs natural behaviour [51]. That these pig production issues did not arise during the discussions may be construed as a lack of concern, but it is more likely, given the low level of knowledge reported, that these practices are largely unknown to the consumer. Future research could seek to identify consumers’ interest in, and preferred methods of addressing these FAW knowledge gaps, e.g., information campaigns or farm open days or visits.

### 4.3. Policy Implications 

The current study offers insights for policies on public engagement efforts on FAW and for policies on food labelling and quality assurance schemes. Options for animal welfare labelling, beyond the current obligatory egg labelling system, are currently being considered at the EU (Farm to Fork) and National Level (Department of Agriculture, Food and the Marine, 2021–2025). Labelling may serve as an important platform for disseminating product welfare information and lessening the gap between consumers and agriculture. Information received from food packaging has been shown to be the most important criteria when making purchasing decisions [52]. Given that the current study indicates a public appetite for more information on FAW, at the same time that they feel increasingly disconnected from farming and food production, food labelling, and quality assurance schemes directly focused on FAW should, in theory, be well received. Conditions for effective front-of-pack food labelling include (i) an awareness and knowledge of the issue pertaining to the credence attribute being communicated on the label; and (ii) consumer demand and motivation to purchase the product based on that credence attribute. The manner in which food labels communicate the issues of ‘farm animal welfare’ would require significant consideration, given that the current study indicates consumers may define and conceptualise FAW differently to the farming and research sector. Future studies exploring the framing impacts of different types of FAW food labels would be of value to better inform policies considering the roll-out of FAW labelling schemes. 

Prior to implementing a food label dedicated to FAW, there needs to be consideration given to how best to ensure integrity and trust in that labelling system. The current findings indicate a lack of public trust around labelling. The perception of higher FAW in one’s own country and the preference for domestically produced food, found within this study, has been found in a large number of studies [14,52]. However, as seen within the discussions, confusion around standards for FAW on the IOI contributed to a lack of trust, which has also been seen in wider research [53]. A lack of trust was also noted in earlier research in Ireland [12], leaving consumers at that time less likely to believe animal welfare claims. Participants in this study felt that more transparency within the agricultural sector would increase their trust. As people increasingly get information in different ways, and as the public becomes more exposed to different forms of information through online and social media, there is now intense scrutiny of labels–what they say, what they mean, and how much they can be trusted. Within Ireland, the importance of building trust in meat production through transparency has been previously identified through consumer trend research, stating “truth tastes better” [54]. Prior to the introduction of any quality assurance or labelling scheme, the perspective of the consumer must be considered. In order to ensure that a scheme is meaningful and impactful, they must meet the expectation of the consumer and make certain that consumers fully comprehend the role that governments and the agricultural industry have in these schemes [31,55]. Further, any labelling needs to be underpinned by a good quality assurance scheme [31] developed from a strong evidence base [55]. Animal-based welfare indicators are regarded by animal welfare scientists as the most valid method of evidencing the welfare of animals on farms [56], and so should be integrated into any developing quality assurance scheme and communicated to the consumer. A high degree of transparency will be necessary to acquire the trust of the modern consumer.

Research has shown that access to information and perception of welfare labelling significantly influences behavioural willingness to purchase higher animal welfare food [57]. However, as was seen in this study and elsewhere [58], a gap often exists between the concerns of the citizen and its translation into shopping behaviour as a consumer. Despite participants acknowledging that they have the power to improve FAW on farms, we still see from the findings on sector-specific concerns that, with the exception of eggs, concerns about welfare may not always translate into buying behaviour. Within the focus groups, priority was given to price, taste, and perceived quality in their decision making when buying animal products. The extra cost of higher FAW products was seen to be the largest barrier to purchasing animal welfare-friendly products and is consistent with the wider research [59,60]. It must also be considered that this citizen-consumer gap currently exists in a setting of low knowledge of farming and FAW and confusion and mistrust of standards and labels. The current study supports high information insufficiency–whereby the public perceive that they currently lack adequate information on FAW and desire more. Public engagement and education efforts would be well placed to address these information and knowledge gaps. Given that consumers self-report low knowledge on the subject of FAW, alongside the lack of awareness shown about hot topic welfare issues being discussed in the agricultural sector, it is evident that a vacuum of information exists relating to food production and FAW, which is contributing to the consumer disconnect. The continued increase in urbanisation is resulting in ever-reducing amounts of young people having any direct experience of farming or awareness of how food is produced. At present, consumers’ main source of information on FAW is mainstream media and social media. This risks leaving consumers vulnerable to the effect of misinformation related to topics of animal food production. A strong argument exists for an increased focus on topics related to where food comes from and about how animals are farmed into the school curriculum [61]. This may allow for system change in that consumers with knowledge and motivation are more likely to support responsible consumerism trends (i.e., FAW food labelling).

## 5. Conclusions

Central to the findings of the current survey is the sector-specific perceptions of FAW held by consumers on the IOI. The difference in sectors can be summarised as follows. On the island of Ireland, cows and cattle are viewed to be part of a pasture-based, extensive system with high public visibility resulting in positive perceptions of welfare. Conversely, poultry and pig farms are viewed as intensive, having low public visibility with issues relating to housing and outdoors access. However, across all sectors, positive public perceptions of FAW appear to be increasing over time. At the same time, high information insufficiency exists–whereby the public feels uninformed and eager to seek out additional information on FAW. Labelling can help attend to perceived public information gaps. However, any labelling scheme must be underpinned by a transparent and evidence-based quality assurance scheme to build public trust. Public engagement efforts, which seek to not only educate, but also empower the public with information on farming and food production, will be a vital strand required to support any future labelling strategies on FAW.

There are a number of limitations to note in the current study. Survey research requires participants to self-report their perceptions and attitudes, and thus, a bias of social desirability arises. Furthermore, a recruitment bias can occur in that participants who hold existing interest in the topic may be more predisposed to take part in the studies. By using a mixed-methods approach, it is hoped that such limitations are balanced by gaining a holistic and triangulated insight into consumer perceptions of FAW. The survey reflected the views of a large sample of the general population, representative of key demographics, thus providing rigour to the findings. The focus groups offered a rich understanding of consumers’ perceptions and beliefs regarding FAW. Future research strands include investigating the gaps that are known to exist between citizen views (as measured in the current study) and actual consumer behaviour (e.g., purchasing of welfare-related food products). 

## Figures and Tables

**Table 1:** Survey sample characteristics.

Group		Location	Recruitment Method
1.	Seniors urban	Belfast City	Convenience
2.	Seniors rural	Co. Tyrone	Convenience
3.	Young adult urban	Dublin City	Convenience
4.	Young adult rural	Co. Donegal	Convenience
5.	Parent urban	Co. Galway	Community Groups
6.	Parent rural	Co. Donegal	Convenience
7.	Vegetarians urban	Belfast City	Posters
8.	Middle-aged mixed	Co. Galway	Flyers
9.	Mixed-age female	Co. Galway	Convenience/posters
Inclusion criteria for all groups: Eats meat/dairy regularly (excluding Group 7) Not farmers Mainly responsible for the food purchase and preparation in the household			

**Table 2:** Survey-based consumer perceptions (*n* = 972) of farm animal welfare in the Republic of Ireland (ROI) and Northern Ireland (NI) across five production sectors ^a^.

	Beef			Poultry Meat			Poultry Eggs			Pork			Dairy		
	ROI	NI	Total	ROI	NI	Total	ROI	NI	Total	ROI	NI	Total	ROI	NI	Total
Very poor	11 (1.6%)	4 (1.4%)	15 (1.5%)	47 (6.8%)	5 (1.8%)	52 (5.3%)	32 (4.7%)	4 (1.4%)	36 (3.7%)	26 (3.8%)	6 (2.1%)	32 (3.3%)	6 (0.9%)	3 (1.1%)	9 (0.9%)
Poor	24 (3.5%)	5 (1.8%)	29 (3%)	94 (13.7%)	17 (6%)	111 (11.4%)	81 (11.8%)	17 (6%)	98 (10.1%)	57 (8.3%)	17 (6%)	74 (7.6%)	21 (3.1%)	5 (1.8%)	26 (2.7%)
Moderate	101 (14.7%)	34 (11.9%)	135 (13.9%)	196 (28.5%)	63 (22.1%)	259 (26.6%)	165 (24%)	52 (18.2%)	217 (22.3%)	153 (22.3%)	52 (18.2%)	205 (21.1%)	109 (15.9%)	29 (10.2%)	138 (14.2%)
Good	242 (35.2%)	96 (33.7%)	338 (34.8%)	183 (26.6%)	87 (30.5%)	270 (27.8%)	216 (31.4%)	87 (30.5%)	303 (31.2%)	245 (35.7%)	89 (31.2%)	334 (34.4%)	237 (34.5%)	110 (38.6%)	347 (35.7%)
Very good	252 (36.7%)	107 (37.5%)	359 (36.9%)	95 (13.8%)	66 (23.2%)	161 (16.6%)	128 (18.6%)	84 (29.5%)	212 (21.8%)	107 (15.6%)	68 (23.9%)	175 (18%)	259 (37.7%)	105 (36.8%)	364 (37.4%)
I don’t know	57 (8.3%)	39 (13.7%)	96 (9.9%)	72 (10.5%)	47 (16.5%)	119 (12.2%)	65 (9.5%)	41 (14.4%)	106 (10.9%)	99 (14.4%)	53 (18.6%)	152 (15.6%)	55 (8%)	33 (11.6%)	88 (9.1%)

^a^ Thinking about farms in Ireland <Northern Ireland>, how do you rate the animal welfare conditions in the following sectors? (*n* = 972).

**Table 3:** Survey-based consumer perceptions (*n* = 972) in the Republic of Ireland (ROI) and Northern Ireland (NI) of change in farm animal welfare over last ten years across five production sectors ^a^.

	Beef			Poultry Meat			Poultry Eggs			Pork			Dairy		
	ROI	NI	Total	ROI	NI	Total	ROI	NI	Total	ROI	NI	Total	ROI	NI	Total
Gotten much worse	6 (0.9%)	2 (0.7%)	8 (0.8%)	19 (2.8%)	9 (3.2%)	28 (2.9%)	10 (1.5%)	5 (1.8%)	15 (1.5%)	13 (1.9%)	5 (1.8%)	18 (1.9%)	5 (0.7%)	4 (1.4%)	9 (0.9%)
Gotten somewhat worse	25 (3.6%)	13 (4.6%)	38 (3.9%)	74 (10.8%)	13 (4.6%)	87 (9%)	59 (8.6%)	9 (3.2%)	68 (7%)	58 (8.4%)	17 (6%)	75 (7.7%)	27 (3.9%)	10 (3.5%)	37 (3.8%)
Is about the same	170 (24.7%)	72 (25.3%)	242 (24.9%)	186 (27.1%)	73 (25.6%)	259 (26.6%)	177 (25.8%)	71 (24.9%)	248 (25.5%)	193 (28.1%)	79 (27.7%)	272 (28%)	151 (22%)	68 (23.9%)	219 (22.5%)
Improved somewhat	254 (37%)	98 (34.4%)	352 (36.2%)	226 (32.9%)	97 (34%)	323 (33.2%)	246 (35.8%)	105 (36.8%)	351 (36.1%)	222 (32.3%)	89 (31.2%)	311 (32%)	247 (36%)	101 (35.4%)	348 (35.8%)
Improved a great deal	171 (24.9%)	59 (20.7%)	230 (23.7%)	106 (15.4%)	55 (19.3%)	161 (16.6%)	124 (18%)	53 (18.6%)	177 (18.2%)	114 (16.6%)	45 (15.8%)	159 (16.4%)	192 (27.9%)	69 (24.2%)	261 (26.9%)
I don’t know	61 (8.9%)	41 (14.4%)	102 (10.5%)	76 (11.1%)	38 (13.3%)	114 (11.7%)	71 (10.3%)	42 (14.7%)	113 (11.6%)	87 (12.7%)	50 (17.5%)	137 (14.1%)	65 (9.5%)	33 (11.6%)	98 (10.1%)

^a^ Thinking about farms in Ireland <Northern Ireland>, how do you rate the animal welfare conditions in the following sectors? (*n* = 972).

**Table 4:** Summary of mean difference in consumer perception of farm animal welfare across five production sectors.

	Sector	M (SD)	(1)	(2)	(3)	(4)
(1)	Beef	4.12 (0.92)				
(2)	Chicken	3.45 (1.12)	0.68 **			
(3)	Eggs	3.65 (1.10)	0.48 **	−0.20 **		
(4)	Pork	3.66 (1.02)	0.46 **	−0.21 **	−0.02	
(5)	Dairy	4.16 (0.86)	−0.04	−0.71 **	−0.51 **	−0.50 **

*n* = 775 (listwise deletion). ** Significant at *p* < 0.001. Higher scores = more positive welfare perceptions.

**Table 5:** Summary of mean difference in consumer perception of change in farm animal welfare over the last 10 years across five production sectors.

	Sector	M (SD)	(1)	(2)	(3)	(4)
(1)	Beef	3.87 (0.88)				
(2)	Chicken	3.60 (1.01)	0.27 **			
(3)	Eggs	3.70 (0.94)	0.17 **	−0.11 **		
(4)	Pork	3.62 (0.96)	0.25 **	−0.03	0.08 *	
(5)	Dairy	3.91 (0.90)	−0.04	−0.31 **	−0.21 **	−0.29 **

*n* = 800 (listwise deletion); * significant at < 0.05; ** significant at < 0.001; higher scores = welfare improved.

**Table 6:** Sector-specific perceptions of farm animal welfare identified through consumer focus groups in the Republic of Ireland (*n* = 25) and Northern Ireland (*n* = 16).

Animal Category	Concerns	Level of Concern	Influence on Purchasing Behaviour
Cows	Cow-calf separation (dairy cows) ‘Forced impregnation’ (dairy cows) Quality of life (dairy cows)	Low	No evidence
Chickens	Living conditions Limited access to outdoors Intensiveness	High	Eggs: Yes Poultry meat: No
Pigs	Living conditions Intensiveness Sentience of the animal Lack of visibility/transparency	High	No evidence

**Table 7:** Comparison of study findings with the Eurobarometer (2005) by combining negative response scores (*) (**).

			Production System		
	Location	*n*	Egg	Dairy	Pork
Eurobarometer 229 (2005) *	UK	1322	58%	13%	27%
	Ireland	997	47%	12%	32%
Current study **	Island of Ireland	972	15%	4%	16%

* Participants were asked to answer “In general, how would you rate the welfare/protection of the following farmed animals?” on a 4 point scale [Very Bad, Fairly Bad, Fairly Good, Very Good]. Displayed here are combined responses for “Very Bad + Fairly Bad”. ** Participants were asked to answer “Thinking about farms in Ireland <Northern Ireland>, how do you rate the animal welfare conditions in the following sectors?” on a 5 point scale [Very Poor, Poor, Moderate, Good, Very Good]. Displayed here are combined responses for “Very poor + Poor”.

## Data Availability

Data is available from the authors upon request.

## Supplementary Materials

The following are available online at https://www.mdpi.com/article/10.3390/ani12020185/s1, Table S1: Survey sample characteristics; Table S2: Demographic profile of focus group participants.
