# Supplementary material for: Current Consumer Perceptions of Animal Welfare across Different Farming Sectors on the Island of Ireland
Source: Animals (Basel). 2022 Jan 13;12(2):185. doi: 10.3390/ani12020185 (PMC8773355; doi:10.3390/ani12020185)
Supplement: Supplementary file 1 [file animals-12-00185-s001.zip › animals-1489339-supplementary.pdf]

Table S1. Survey sample characteristics.

Characteristic	*n* = 972
Age (years)	
18–24	65 (6.7%)
25–34	201 (20.7%)
35–44	240 (24.7%)
45–54	183 (18.8%)
55–64	151 (15.5%)
65+	132 (13.6%)
Gender	
Male	446 (45.9%)
Female	526 (54.1%)
Country of residence	
Northern Ireland	285 (29.3%)
Republic of Ireland	687 (70.7%)
Area of residence	
Urban City	212 (21.8%)
Urban Town	220 (22.6%)
Suburban	265 (27.3%)
Rural Village	118 (12.10%)
Rural Countryside	157 (16.2%)
Social Class [1]	
ABC1F+	549 (56.5%)
C2DEF-	423 (43.5%)
Education status	
Primary or no formal education	9 (0.9%)
Lower secondary	61 (6.3%)
Higher secondary	205 (21.1%)
Post Leaving Certificate/A-Level	81 (8.3%)
Third level	594 (61.1%)
Not stated	22 (2.2%)
Dietary Preferences	
Vegetarian	40 (4.1%)
Partial vegetarian	94 (9.7%)
Meat eater	817 (84.1%)
Other dietary restrictions	21 (2.2%)

[1] Social class is categorised by the Central Statistics Office based on the level of skill and educational attainment of one’s occupation. ABC1F+ = Professional, managerial, and technical; C2DEF- = Skilled or semi-skilled manual and unskilled.

Table S2. Demographic profile of focus group participants.

Characteristic	1	2	3	4	5	6	7	8	9	Total
	(*n* = 6)	(*n* = 5)	(*n* = 4)	(*n* = 4)	(*n* = 5)	(*n* = 4)	(*n* = 5)	(*n* = 3)	(*n* = 5)	(*n* = 41)
Gender										
Male	0	0	0	4	0	1	2	2	0	9
Female	6	5	4	0	5	3	3	1	5	32
Age										
18–35	0	0	4	4	5	0	4	0	0	17
36–55	0	1	0	0	0	4	1	3	1	10

56+	6	4	0	0	0	0	0	0	4	14
Region										
ROI	0	0	4	4	5	4	0	3	5	25
NI	6	5	0	0	0	0	5	0	0	16
Children										
< 12	0	0	0	0	5	4	2	2	0	13
None < 12	6	5	4	4	0	0	3	1	5	28
Education level[a]										
Secondary	1	2	0	0	5	0	0	1	1	10
Graduate	3	0	1	2	0	4	1	0	2	13
Post-graduate	2	1	3	2	0	0	4	2	2	16
House income[b]										
< EUR 25,000	3	5	0	0	5	2	3	0	1	19
EUR 25,000–50,000	2	0	4	3	0	1	1	2	2	15
> EUR 50,000	0	0	0	1	0	1	1	1	2	6
Pet ownership										
Yes	5	4	1	1	4	3	3	2	1	24
No	1	1	3	3	1	1	2	1	4	17
Farm Visits										
Regular access	2	0	4	2	0	0	0	0	1	9
Visited in past	4	4	0	2	2	4	4	3	4	27
Never been	0	1	0	0	3	0	1	0	0	5

Groups: 1. Senior Urban, 2. Senior Rural, 3. Young Adult Urban, 4. Young Adult Rural, 5. Parent Urban, 6. Parent Rural, 7. Vegetarians, 8. Mid-aged mixed, 9. Mixed-aged Rural. [a] Missing data (n = 2); [b] Missing data (n = 1)
